# Endoscopic sinus surgery outcomes in patients with chronic rhinosinusitis and immunoglobulin deficiencies

**DOI:** 10.1186/s40463-023-00648-3

**Published:** 2023-06-29

**Authors:** Shireen Samargandy, Elysia Grose, Jonathan Yip, John M. Lee

**Affiliations:** 1 Division of Rhinology, Department of Otolaryngology – Head and Neck Surgery, St. Michael’s Hospital, Unity Health Toronto, University of Toronto, 30 Bond Street, 8 Cardinal Carter, Toronto, ON M5B 1W8 Canada; 2 Section of Otolaryngology – Head and Neck Surgery, Department of Surgery, University of Calgary, Calgary, AB Canada

**Keywords:** Immunodeficiency, Endoscopic sinus surgery, Chronic rhinosinusitis, Revision surgery, Immune dysfunction, Nasal polyps, Immunoglobulin replacement therapy

## Abstract

**Background:**

Patients with chronic rhinosinusitis (CRS) and immunoglobulin deficiencies (ID) have more recalcitrant sinonasal disease and a subset of these patients undergo surgical management for their CRS. However, there is a paucity of literature on the surgical outcomes in this patient population and appropriate treatment algorithms for CRS in patients with ID. The objective of this study was to better elucidate the outcomes of endoscopic sinus surgery (ESS) in patients with ID in terms of disease-specific quality-of-life scores and the need for revision surgery.

**Methods:**

A case–control study was performed comparing adult patients with ID and healthy controls that had undergone ESS for CRS. Patients were matched based on age, sex, CRS phenotype, and preoperative Lund-Mackay score. The revision surgery rates, time to revision surgery, and changes in sinonasal outcome tests (SNOT-22) were evaluated.

**Results:**

Thirteen patients with CRS and ID were matched to 26 control patients with CRS. The revision surgery rate for cases and controls was 31% and 12%, respectively, but there was no statistical difference (*p* > 0.05). There was a clinically meaningful reduction in SNOT-22 scores in both groups from the preoperative to postoperative period [mean of 12 points in patients with ID (*p* = 0.323) and 25 points in controls (*p* < 0.001)], however, there was again no significant difference between cases and controls (*p* > 0.05).

**Conclusion:**

Our data suggests that patients with ID have clinically meaningful improvement in SNOT-22 scores after ESS but may have higher revision rates than immunocompetent patients with CRS. ID are rare disease entities, thus most attempts at studying this cohort would be limited by sample size. Further homogenous data on immunoglobulin deficient patients is required for future meta-analysis to better understand the impact of ESS in patients with ID.

**Supplementary Information:**

The online version contains supplementary material available at 10.1186/s40463-023-00648-3.

## Introduction

The relationship between recalcitrant chronic rhinosinusitis (CRS) and immunoglobulin deficiencies (ID) has been well established with some reports demonstrating that up to 54% of patients with refractory CRS have an underlying ID [1–3]. Given the rarity of this condition, it is unsurprising that the treatment of CRS in patients with ID has been consistently identified as an area requiring further research by several clinical guidelines [4, 5]. This patient population is compared to other types of immunodeficiency given the congenital nature of the disease and the unique treatment options available to them such as immunoglobulin replacement therapy (IRT) [6].

Given the recalcitrant nature of CRS in patients with ID, many patients eventually require endoscopic sinus surgery (ESS). The evidence on the outcomes of ESS in this population is lacking. This has many reasons, including the propensity for exclusion from large cohorts, the heterogeneity of the types of immunodeficiencies in reported cohorts, as well as  the use of non-validated clinical outcome measures. In a systematic review performed by the authors of this study, ESS was found to be beneficial for symptom control in patients with immunodeficiency [7]. Based on the systematic review and following literature review, we did not identify any studies investigating the outcomes of ESS in a homogenous group of patients with ID without conglomeration with other types of immunodeficient states; such as solid organ transplant recipients or patients with human immunodeficiency virus (HIV) infection [7]. Thus, the primary objective of this study was to evaluate the sinonasal outcomes after ESS in patients with ID as defined by the change in quality-of-life (QoL) scores and need for revision surgery.

## Methods

### Study design

A retrospective case–control study of adult patients (18 years of age and older) who underwent ESS for CRS between 2009 and 2019 was conducted. Patients were identified from the senior author’s (J.M.L) surgical database and their electronic records were reviewed. Approval of this study was granted by the Unity Health Toronto Research Ethics Board.

### Selection of cases and controls

Cases were defined as patients with ID and CRS that underwent ESS. Diagnosis of ID was made by a clinical immunologist and based on reduced serum levels of one or more of the following immunoglobulins: IgA, IgE, IgG, and IgG I-V subclasses. Patients with primary immunodeficiencies including common variable immunodeficiency (CVID) and those with isolated IDs were included in the study [8]. Patients with both chronic rhinosinusitis with nasal polyps (CRSwNP) and chronic rhinosinusitis without nasal polyps (CRSsNP), as defined by the American Academy of Otolaryngology-Head and Neck Surgery guidelines, were included [9].

Controls were selected if they underwent ESS for CRSsNP or CRSwNP and did not have ID. We excluded patients with recurrent acute rhinosinusitis, cystic fibrosis, ciliary dysplasia, aspirin exacerbated respiratory disease, granulomatosis with polyangiitis, and allergic fungal sinusitis. Patients with clinically significant acquired immunodeficiencies such as uncontrolled HIV infection, chronic immunosuppressive medication use, and hematologic malignancies were excluded as well.

### Data collected

Information on age, sex, comorbidities, CRS phenotype, type of immunodeficiency, extent of surgery, and revision status was collected. All cases and controls had preoperative sinus computed tomography (CT) scans available. The Sino-Nasal Outcome Test-22 (SNOT -22) survey was administered at each clinic visit. Preoperative Lund-MacKay scores (LMS) were scored by a blinded, senior Otolaryngology-Head and Neck Surgery resident (S.S.). Immunoglobulin deficiencies and whether IRT (for example, intravenous or subcutaneous immunoglobulin) was required was determined through documentation of a consulting immunologist. The IRT regimens were recorded when applicable.

### ESS and postoperative management

The extent of ESS was tailored to the degree of disease and the involved sinuses using the Messerklinger technique. Complete ESS was defined as maxillary antrostomies, total ethmoidectomies, sphenoidotomies, frontal sinusotomies, and nasal polypectomies if polyps were present. Limited ESS was defined as any ESS that did not fit the definition of complete ESS [10]. Select patients underwent concurrent endoscopic septoplasty if clinically indicated. Revision ESS was defined as any revision surgery, either complete or limited ESS, by the senior author (J.M.L.).

Postoperatively, these patients were prescribed clarithromycin 500 mg twice daily for two weeks if not allergic and instructed to continue with high volume saline nasal rinses at least twice daily. Prednisone was prescribed depending on the burden of nasal polyps. Intranasal steroids were restarted 2 weeks post-surgery.

### SNOT-22 scores

All available SNOT-22 scores for each patient were collected. In addition to total SNOT-22 scores, the individual ratings from each of the twenty-two questions were collected. The 22 items in the SNOT-22 survey were then subclassified into 5 domains (Additional file 1) [11]. Postoperative SNOT-22 scores were only included in the analysis if they had been completed ≥ 3 months after surgery.

### Matching process and statistical analysis

Descriptive statistics were used to summarize the characteristics of the overall cohort. The ‘Matching’ package in R was used to match cases and controls. The cases were matched to controls based on age (± 10 years), sex, disease status (CRSsNP vs. CRSwNP), and LMS (± 5 points). To assess covariate balance, standardized mean differences (SMD) were examined. A SMD < 0.1 indicated a good balance between the two groups. Means and standard deviations (± SD) were used to report quantitative data. Rate of revision surgery was compared using Fisher’s exact test. Changes in SNOT-22 scores and times to revision surgery were compared using the Mann–Whitney test. *P* values were adjusted (*P*_adj_) using the Holm–Bonferroni method to account for multiple comparisons where applicable. A significance level of 0.05 was used for all tests. All analyses were performed in R version 3.6.3.

## Results

### Patient characteristics

There were 13 cases and 26 controls. The demographic and clinical variables are reported in Table 1. Good balance was observed among the matching variables apart from the LMS (SMD = 0.274). The average difference in Lund MacKay scores between the cases and controls was 0.85 +/− 1.69 points. Based on inspection of the distribution of LMS and clinical judgment, this difference was not considered large enough to be clinically significant. The mean follow-up time for the controls was 24 (± 22.5) months, and the mean follow-up time for cases was 44 (± 36.7) months.

**Table 1:** Characteristics of cases and controls undergoing endoscopic sinus surgery

	Patients with immunoglobulin deficiency (n = 13)	Patients without immunoglobulin deficiency (n = 26)	SMD^a^
Male (n, %)	5 (39)	10 (39)	< 0.001
Age (mean ± SD)	44.2 (15)	43.7 (14)	0.040
Asthma (n, %)	6 (46)	12 (46)	< 0.001
CRSwNP (n, %)	8 (62)	16 (62)	< 0.001
CRSsNP (n, %)	5 (39)	10 (39)	
Preoperative LMS (mean ± SD)	13.8 (± 3.0)	12.9 (± 3.2)	0.274
Follow up time in months (mean ± SD)	44 (± 36.7)	24.0 (± 22.5)	NA
Complete ESS^b^	9 (69)	24 (92)	NA
Limited ESS^c^	4 (31)	2 (8)	NA
Concurrent Septoplasty	2 (15)	7 (27)	NA

*CRS* chronic rhinosinusitis, *CRSsNP* chronic rhinosinusitis withoutt nasal polyps, *CRSwNP* chronic rhinosinusitis with nasal polyps, *LMS* Lund MacKay score, *SD* standard deviation, *SMD* standardized mean difference, *ESS* endoscopic sinus surgery

^a^A SMD < 0.1 indicated a good balance between the two groups

^b^Complete ESS was defined as maxillary antrostomies, total ethmoidectomies, sphenoidotomies, frontal sinusotomies, and nasal polypectomies if polyps are present

^c^Limited ESS was defined as any ESS that did not fit the definition of complete ESS

Among the cases, 4 (31%) had common variable immunodeficiency, 5 (38%) had IgG deficiency, 3 (23%) had unspecified hypogammaglobulinemia, and 1 (8%) had IgE deficiency. Six patients (46%) with ID received IRT. Three patients were on monthly doses of 30–40 g of intravenous immunoglobulin (IVIG). One patient received 45 g of IVIG every 2–3 weeks. Two patients were on subcutaneous immunoglobulin injections ranging from 6 to 8 g per week. Of the 6 patients who received IRT, 50% of them had CRSwNP and 67% of them had CVID. The IRT was indicated in these patients as 4/6 (67%) experienced recurrent pneumonia, whereas 2/6 (33%) experienced recurrent sinus infections and gastrointestinal infections. Two (33%) of the patients on IRT required revision FESS.

### SNOT-22 scores

Ten patients with ID and 21 control patients had completed preoperative and postoperative SNOT-22 questionnaires. Preoperatively, the mean score among patients with ID was 54.7 (± 25.3) and among control patients, the mean score was 51.0 (± 22.9). Postoperatively, the mean score for patients with ID and control patients was 42.6 (± 24.8) and 26.0 (± 22.6), respectively. Changes in total and subdomain SNOT-22 scores from pre- to post-surgery are presented in Table 2. There were no significant changes in the mean preoperative and postoperative score in patients with ID (*p* = 0.323). However, there were significant differences between preoperative SNOT-22 and postoperative SNOT-22 scores in the control group (*p* < 0.001).

**Table 2:** Comparison of preoperative and postoperative SNOT-22 scores between cases and controls

	Mean change^a^ in SNOT-22 for patients with immunoglobulin deficiencies (SD) (n = 10)	Mean change^a^ in SNOT-22 for controls (SD) (n = 21)	*P* value
Total SNOT-22 score	− 12.1 (26.0)	− 25.0 (13.4)	0.218
Rhinologic symptoms domain	− 3.3 (7.3)	− 9.1 (5.3)	0.126
Extranasal rhinologic symptoms domain	− 1.4 (4.3)	− 4.1 (2.9)	0.145
Ear/facial symptoms domain	− 1.7 (4.8)	− 4.7 (4.0)	0.294
Psychological dysfunction domain	− 5.3 (11.2)	− 7.2 (6.3)	0.580
Sleep dysfunction domain	− 2.8 (5.6)	− 4.0 (4.5)	0.580

^a^These values represent the mean of the change in SNOT-22 score for each individual patient

*IQR* interquartile range, *SNOT-22* sinonasal outcomes test-22 items

### Revision rates

Four cases and 3 controls underwent revision surgery resulting in a revision surgery rate of 31% and 12%, respectively (*p* = 0.194). Table 3 summarizes the characteristics of both the cases and controls who did and did not require revision surgery. The mean time to revision surgery for cases was 41.5 (± 22.9) months and the mean time for controls was 46.7 (± 31.4) months (*p* = 1.00).

**Table 3:** Comparison of cases and controls who did and did not undergo revision surgery

	Revision surgery (Cases) (N = 4)	No revision surgery (Cases) (N = 9)	Revision surgery (Controls) (N = 3)	No Revision surgery (Controls) (N = 23)
Male (n, %)	2 (50)	3 (33)	1 (33)	8 (35)
Age (mean ± SD)	34.8 ± 8.6	48.4 ± 15.9	45.3 ± 20.0	43.4 ± 13.3
Asthma (n, %)	2 (50)	4 (44)	1 (33)	11 (48)
CRSwNP (n, %)	4 (100)	4 (44)	2 (67)	14
CRSsNP (n, %)	0 (0)	5 (56)	1 (33)	9
Preoperative LMS (mean ± SD)	14 ± 2.2	13.7 ± 3.4	13.3 ± 6.1	12.7 ± 2.9
Complete ESS^a^ (%)	4 (100)	7 (78)	3 (100)	22
Limited ESS^b^ (%)	0 (0)	2 (22)	0 (0)	1

*SD* standard deviation, *ESS* endoscopic sinus surgery, *LMS* Lund Mackay score, *CRSsNP* chronic rhinosinusitis without nasal polyps, *CRSwNP* chronic rhinosinusitis with nasal polyps

^a^Complete ESS was defined as maxillary antrostomies, total ethmoidectomies, sphenoidotomies, frontal sinusotomies, and nasal polypectomies if polyps are present

^b^Limited ESS was defined as any ESS that did not fit the definition of complete ESS

## Discussion

The lack of guidelines for the management of CRS in patients with immunodeficiencies has led to clinical uncertainty and highly variable practice patterns [12]. CRS in patients with ID has been identified as an area where further evidence is needed in both the recent International Consensus Statement on Allergy and Rhinology for Rhinosinusitis and the European Position Paper on Rhinosinusitis [4, 5]. Furthermore, the impact of underlying ID on sinus surgery outcomes remains unclear [3, 7]. Thus, there is a compelling need for further research in this area, specifically for patients with ID as the pathophysiology of their immunodeficiency is unique, and they are candidates for other modalities of treatment, primarily IRT.

The limited available research on ESS in adult patients with immunodeficiencies suggests that they may experience similar benefit in terms of QOL scores when compared to immunocompetent patients, however no data solely addresses patients with ID [2, 7, 13, 14]. Khalid et al. conducted a case control study of ESS outcomes in patients with immune dysfunction (that included both immunodeficiencies and autoimmune conditions) and found that both cases and controls had similar improvements in QOL scores (Chronic Sinusitis Survey and Rhinosinusitis Disability Index) after ESS [13]. A study by Miglani et al. found a median reduction of 17 points in SNOT-22 at 6 months after ESS in 21 patients with immunodeficiency[15]. However, they did not define the types of immunodeficiencies included. In our study, the mean change between preoperative and postoperative scores was a 12-point decrease in SNOT-22 score in the patients with ID and a 25-point decrease in the controls. Although patients with ID experienced clinically significant improvement in SNOT-22, our study did not detect a statistically significant difference between their preoperative and postoperative scores [16–18]. Unsurprisingly, there was improvement in SNOT-22 postoperatively for controls (*p* < 0.001). There was also no statistically significant difference in SNOT-22 improvement between cases and controls (*p* = 0.323).

This study also evaluated the revision surgery rates and found a 31% revision surgery rate in the cases and a 12% rate in the controls. When compared to other studies assessing revision surgery rates in patients with CRS and no immunodeficiency, the revision surgery rates ranged from 4 to 16% with asthma, aspirin exacerbated respiratory disease, and nasal polyps having higher rates of revision surgery [15, 19–21]. Miglani et al. [15] reported a 14% revision surgery rate for patients with immunodeficiency, however their overall mean follow up time was 28 months whereas the present study had a 44 month mean follow up time for patients with ID. Other studies reporting on revision surgery rates in patients with immunodeficiency included exclusively patients with HIV and reported rates of 0–15% [22, 23]. However, these patients have a different pathophysiology underlying their immunodeficiency, and measures to assess disease control (such as CD4 count and viral load) were inconsistently reported in these studies. The revision surgery rate among patients with ID in this study is likely higher than patients without immunodeficiency based on the available evidence both in our study and the available literature; however, this did not meet statistical significance (*p* = 0.194).

The mean time to revision surgery was similar between both the cases and controls at 42 months and 47 months, respectively, which is comparable to other literature reporting on time to revision surgery in CRS [19, 20, 24, 25]. The present study found no significant differences between the time to revision surgery between cases and controls.

We acknowledge several limitations. Firstly, the group of patients with ID remains heterogenous to some degree as both patients with CVID and isolated immunoglobulin deficiencies were included. Additionally, the controls used in this study were not specifically screened for ID as this is not part of our routine practice and thus, could have had subclinical ID. Because ID is a rare clinical entity, the small sample size in this study could have impacted our ability to detect statistically significant differences between the cases and controls. Moreover, there was heterogeneity in the extent of surgery and postoperative regimens which were not directly accounted for in the matching model but were approximated through matching on LMS and polyp status. Although disease severity was quantified by preoperative LMS, this study did not include endoscopy scores, frequency of acute sinus or pulmonary exacerbations, or use of antibiotics. Given that patients with ID are more susceptible to infection, the impact of sinus surgery on the frequency and severity of acute exacerbations is an area which certainly deserves further investigation. There is preliminary evidence to suggest that patients with ID had a reduction in the mean number of antibiotics prescribed for sinopulmonary infections and LM scores after ESS [26]. Unfortunately, there was no reliable way to determine the number of acute exacerbations or antibiotic prescriptions using the electronic medical record used to collect our data. Furthermore, 6 cases received IRT and the impact of this was not accounted for in our study. Finally, the controls had a shorter follow up time than the cases (24 months vs. 44 months) and thus we may have missed additional revision surgeries in the control group that were indicated later. However, there is evidence which suggests that almost half of revision surgeries are performed within 1 year of the initial surgery and thus, the follow up time for controls in the current study would have been adequate to include most patients who required revision surgery [20]. Despite these limitations, this study represents one of the largest studies on ESS outcomes in immunodeficient patients, and the first to report surgical outcomes in a homogenous cohort of patients with ID. In addition, this study addresses several of the limitations in the existing literature on this patient population including using standardized QoL measures, clearly defining the extent of surgery, and clearly defining the immunodeficient patient population [7]].

## Conclusion

This study represents one of the first and largest case–control studies to investigate ESS outcomes in patients with ID. The results of this study suggests that patients with ID experience clinically meaningful improvement in SNOT-22 scores after ESS, but may have higher revision rates than other patients with CRS. However, given the limitations of this study, further research is needed to clarify the impact of ESS in this patient population and specifically on acute exacerbations of sinopulmonary disease and the impact of IRT on surgical outcomes in this patient population.

## Supplementary Information

**Additional file 1.** SNOT-22 domains and survey items.

## Data Availability

The datasets used and analysed during the current study are available from the corresponding author on reasonable request.
