# Supplementary material for: Endoscopic sinus surgery outcomes in patients with chronic rhinosinusitis and immunoglobulin deficiencies
Source: J Otolaryngol Head Neck Surg. 2023 Jun 29;52:43. doi: 10.1186/s40463-023-00648-3 (PMC10308710; doi:10.1186/s40463-023-00648-3)
Supplement: Supplementary file 1 — Additional file 1. SNOT-22 domains and survey items. [file 40463_2023_648_MOESM1_ESM.docx]

**Supplementary Material**

**Table 1: SNOT-22 domains and survey items**

| **SNOT-22 Domain** | **Survey Items** | **Score range** |
| --- | --- | --- |
| **Rhinologic Symptoms** | Need to blow nose  Sneezing  Runny nose  Nasal obstruction  Loss of sense of smell or taste  Thick nasal discharge | 0-30 |
| **Extra-Nasal Rhinologic Symptoms** | Cough  Post-nasal discharge  Thick nasal discharge | 0-15 |
| **Ear/Facial Symptoms** | Sneezing  Ear fullness  Dizziness  Ear pain/pressure  Facial pain/pressure | 0-25 |
| **Psychological Symptoms** | Waking up tired  Fatigue during the day  Reduced productivity  Reduced concentration  Frustrated/ restless/irritable  Sad  Embarrassed | 0-35 |
| **Sleep Dysfunction** | Difficulty falling asleep  Waking up at night  Lack of a good night’s sleep  Waking up tired  Fatigue during the day | 0-25 |

**SNOT-22: Sinonasal Outcome Test-22**
